# Computational Mapping of Complex Traits in the Mouse

**DOI:** 10.1371/journal.pbio.0020425

**Published:** 2004-11-09

The past few years have seen an explosion of data on the mouse genome, widely hailed as a guidebook to the genetic origins of human disease. Scientists are particularly interested in charting the location of SNPs—single nucleotide polymorphisms—throughout the genome. SNPs are DNA sequence variations, or mutations, that change a single nucleotide in the genome, replacing a cytosine (C) base, say, with thymine (T). Many SNPs, even those found within genes, have no functional effect. Others, however, can increase risk of specific diseases or alter a person's response to pathogens and drugs. Whether or not they are involved directly in a disease, SNPs are attractive markers for population studies aimed at identifying the multiple genes underlying complex diseases like diabetes and cancer.

Extensive data exist on multiple inbred strains of mice linking their genetic makeup (genotype) to physical traits (phenotype), and scientists have used these data to guide investigations of gene function and disease. Many data have been gathered by crossing mouse strains and painstakingly analyzing their progeny, to tease out the relative contributions different genes make to pathogenesis. But these efforts take time. Investigators would greatly benefit from high-throughput methods to scan the mouse genome and flag markers for candidate disease genes. In 2001, Andrew Grupe et al. introduced an “in silico” (computational) approach to do that very thing. The method scanned mouse SNP data to home in on chromosomal areas regulating complex traits and reduce the time needed to analyze mouse disease models from “many months” to “milliseconds.”

For a number of reasons, however, it wasn't clear whether in silico mapping could deliver on its promise. For one thing, the density of SNP maps was sufficient to provide meaningful markers for only a few mouse strains, and phenotype information was lacking for many strains. In a new study, Tim Wiltshire and colleagues have addressed these limitations by mapping nearly 11,000 SNP probes to 48 mouse strains. They have also been able to use this dataset for in silico mapping to predict genomic regions with functionally important phenotypes.

Wiltshire and colleagues first show that their method can predict the genomic location of a Mendelian trait (controlled by a single gene), in this case coat color, which the authors acknowledge is a “minimum requirement for a viable in silico mapping method.” They go on to map complex “quantitative” traits (controlled by differential contributions from multiple genes at what are called quantitative trait loci, or QTLs)—gallstone development and plasma levels of high-density lipoprotein cholesterol—and find that their predictions fall in line with loci identified by traditional mouse disease studies. Noting a high correlation between QTLs predicted in silico and those identified experimentally, the authors argue that loci predicted using this method are “very likely to be biologically relevant.”[Fig pbio-0020425-g001]

**Figure pbio-0020425-g001:**
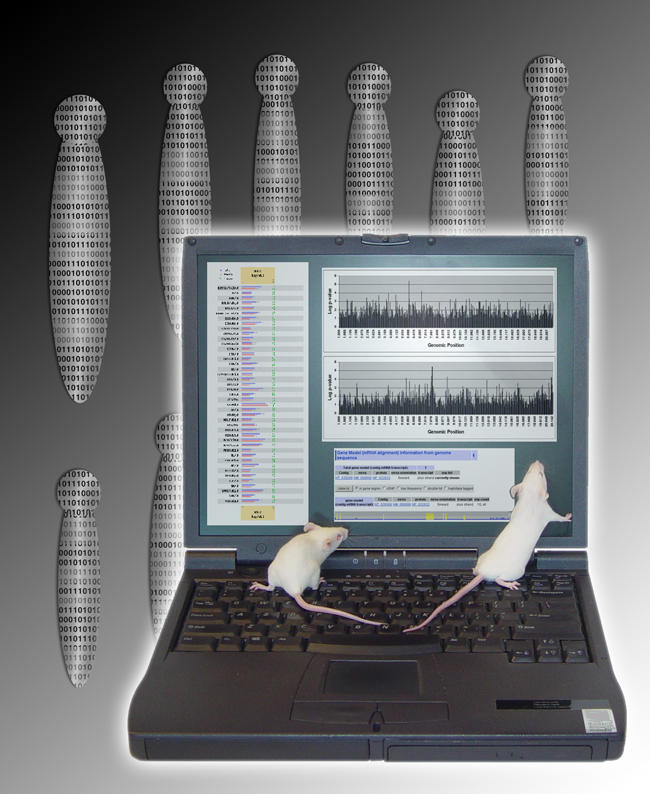
In silico mapping for mouse genetics

Wiltshire and colleagues are careful to point out that in silico mapping is meant to complement, not replace, traditional gene mapping models. After all, computers are no match for living organisms in modeling the subtleties inherent in biological reactions. But this approach is a good starting point for identifying significant genomic areas in a new strain. And as new strains are genotyped and phenotyped, and refinements are made to the SNP database, the robustness of this method should only get better.

